# Rafoxanide Induces Immunogenic Death of Colorectal Cancer Cells

**DOI:** 10.3390/cancers12051314

**Published:** 2020-05-21

**Authors:** Antonio Di Grazia, Federica Laudisi, Davide Di Fusco, Eleonora Franzè, Angela Ortenzi, Ivan Monteleone, Giovanni Monteleone, Carmine Stolfi

**Affiliations:** 1 Department of Systems Medicine, University of Rome “Tor Vergata”, 00133 Rome, Italy; adigrazia2000@yahoo.it (A.D.G.); federica.laudisi@uniroma2.it (F.L.); di.fusco@med.uniroma2.it (D.D.F.); eleonorafranze@yahoo.it (E.F.); angela.ortenzi@uniroma2.it (A.O.); gi.monteleone@med.uniroma2.it (G.M.); 2 Department of Biomedicine and Prevention, University of Rome “Tor Vergata”, 00133 Rome, Italy; ivan.monteleone@uniroma2.it

**Keywords:** drug repurposing, anti-helmintic, endoplasmic reticulum stress, calreticulin, ATP, HMGB1, drug repositioning, vaccination

## Abstract

Colorectal cancer (CRC) is a major cause of cancer-related death in the world. Emerging evidence suggests that the clinical success of conventional chemotherapy does not merely rely on cell toxicity, but also results from the restoration of tumor immune surveillance. Anti-tumor immune response can be primed by immunogenic cell death (ICD), a form of apoptosis associated with endoplasmic reticulum stress (ERS) induction and the expression/release of specific damage-associated molecular patterns (DAMPs). Unfortunately, a limited number of ICD inducers have been identified so far. The anti-helmintic drug rafoxanide has recently showed anti-tumor activity in different cancer types, including CRC. As such latter effects relied on ERS activation, we here investigated whether rafoxanide could promote ICD of CRC cells. The potential of rafoxanide to induce ICD-related DAMPs in both human and mouse CRC cells was assessed by flow-cytometry, chemiluminescent assay and ELISA. In addition, the immunogenic potential of rafoxanide was assessed in vivo using a vaccination assay. Rafoxanide induced all the main DAMPs (ecto-calreticulin exposure, adenosine triphosphate (ATP)/high mobility group box 1 (HMGB1) release) required for ICD. We observed a marked increase of tumor-free survival among immunocompetent mice immunized with rafoxanide-treated dying tumor cells as compared with sham. Altogether, our data indicate rafoxanide as a bona fide ICD inducer.

## 1. Introduction

Colorectal cancer (CRC) remains the second most-common cause of cancer-related death in the Western world [1], mainly because of the lack of effective therapies in patients with advanced disease. In addition to surgery and immunotherapy, chemotherapy still plays a pivotal role in the treatment of CRC patients. Beyond cancer-cell-intrinsic factors that determine the cytostatic or cytotoxic response, the outcome of chemotherapy is influenced by the host immune system at multiple levels [2]. In the last decade, the functional state of the host immune system has emerged as a main prognostic and predictive factor on the fate of cancer patients treated with conventional or targeted chemotherapies [2]. Consistently, the ability of such drugs to promote the activation of tumor-reactive adaptive immune responses appears as a critical requirement underlying their clinical effectiveness [2,3]. Some cytotoxicants (e.g., anthracyclines, oxaliplatin, radiation therapy, oncolytic viruses) are known to mediate part of their anti-cancer actions through indirect, immune-dependent mechanisms [4,5,6,7,8]. These cytotoxic agents can induce a particular form of apoptosis, termed immunogenic cell death (ICD), which is preceded by pre-mortem stress responses (e.g., calreticulin (CALR) exposure on the cell surface, adenosine triphosphate (ATP) and high mobility group box 1 (HMGB1) release) and that allows the cancer cell to be recognized and handled by the immune system [9]. However, only a limited number of anti-cancer drugs have been identified as ICD inducers, and their approved use is restricted to certain cancer types. Thus, the discovery and/or validation of novel anti-cancer agents that can increase cancer cell immunogenicity is highly desirable.

The anti-helmintic drug rafoxanide is approved by the Food and Drug Administration (FDA) for the veterinary treatment of fascioliasis and some gastrointestinal roundworms [10,11,12]. Concerning the usage of rafoxanide in humans, despite poor evidence, a past study reported the therapeutic use of the drug in a seven-year-old girl affected by fascioliasis [13]. Recently, rafoxanide has been shown to hamper the oncogenic function of the BRAF V600E mutant protein [14], commonly found in melanomas and CRCs and associated with a poorer prognosis for patients [15,16]. In the last two years, rafoxanide has emerged as an anti-tumor agent for different cancer types, both in vitro and in pre-clinical models, acting via multiple mechanisms [17,18,19,20,21]. In particular, we found that rafoxanide restrains the proliferation of CRC cells, but not of normal colonic epithelial cells [21]. Rafoxanide’s anti-mitogenic ability was associated with cyclin D1 protein down-regulation and accumulation of cells in the G0/G1 phase. These effects relied on selective induction of the endoplasmic reticulum stress (ERS) response in CRC cells and were followed by caspase-dependent cell death [21].

As the activation of an ERS response is one of the key events in the course of ICD [22], in this paper, we sought to investigate whether the rafoxanide-induced cell death observed in CRC cells could be immunogenic. We here show that rafoxanide treatment induces pre-mortem CALR exposure in CRC cells, followed by ATP and HMGB1 release. These results and the observation that the vaccination of immunocompetent mice with rafoxanide-treated dying tumor cells prevents subsequent tumor growth indicate rafoxanide as a bona fide ICD-inducing agent.

## 2. Results

### 2.1. Treatment of CRC Cells with Rafoxanide Induces Pre-Apoptotic Exposure of CALR on the Cell Surface

**Figure 1:**
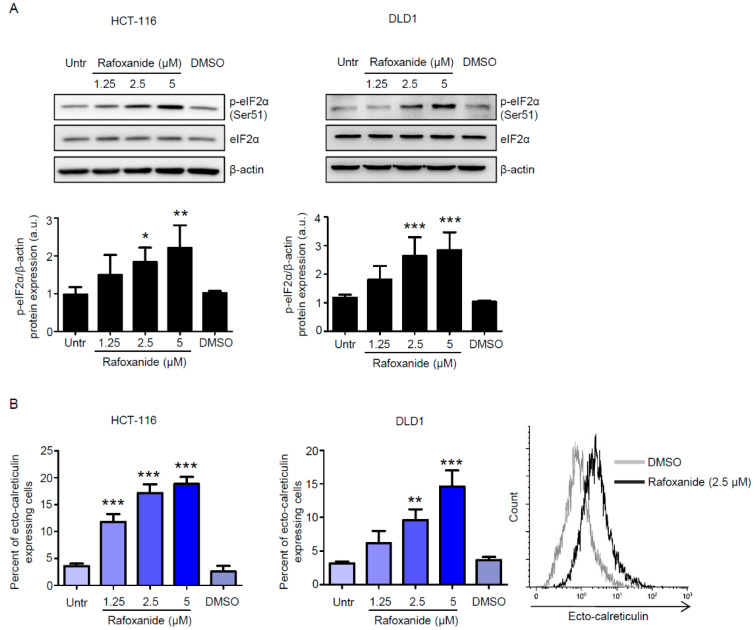
Rafoxanide induces eIF2α phosphorylation and calreticulin exposure on the cell surface in CRC cells. (**A**) Representative Western blotting for p-eIF2α (Ser51) and eIF2α in extracts of HCT-116 and DLD1 cells either left untreated (Untr) or treated with either dimethyl sulfoxide (DMSO) (vehicle) or rafoxanide for 30 min. β-actin was used as loading control. The full blots are available in Figure S1 from Supplementary Materials. Data are representative of four experiments where similar results were obtained. Lower insets: Quantitative analysis of p-eIF2α (Ser51)/β-actin protein ratio in total extracts of HCT-116 and DLD1 as measured by densitometry scanning of Western blots. Values are expressed in arbitrary units (a.u.) and are the mean ± SD of four experiments. Data were analyzed using one-way analysis of variance (ANOVA) followed by Dunnett’s post hoc test. DMSO vs. rafoxanide-treated cells, * *p* < 0.05, ** *p* < 0.01, *** *p* < 0.001. (**B**) Histograms showing the percentage of ecto-calreticulin-expressing HCT-116 and DLD1 cells either left untreated (Untr) or treated with either DMSO or rafoxanide for 6 h. Results indicate the percentage of ecto-calreticulin-expressing cells as assessed by flow-cytometry analysis. Data are expressed as mean ± SD of three experiments. Data were analyzed using one-way analysis of variance (ANOVA) followed by Dunnett’s post hoc test. DMSO vs. rafoxanide-treated cells, ** *p* < 0.01, *** *p* < 0.001. Right inset. Representative histograms showing ecto-calreticulin in HCT-116 treated with either DMSO or rafoxanide as assessed by flow-cytometry.

Endoplasmic reticulum stress (ERS) response, culminating with the phosphorylation of eukaryotic translation initiation factor 2α (eIF2α) on Ser51, is mandatory for cells undergoing ICD to emit all the signals that are required for the initiation of the host immune response [23]. eIF2α phosphorylation is instrumental in the pre-apoptotic exposure of the endoplasmic reticulum chaperone calreticulin (CALR) on the cell surface [7,24], which stimulates the phagocytosis of portions of the dying cancer cell (with the tumor-associated antigen) by the dendritic cells (DCs) [25]. As we recently reported that rafoxanide exerted ERS-dependent selective anti-tumor activity against CRC cells in vitro and in vivo [21], we first assessed whether treatment of CRC cells with increasing doses of rafoxanide—1.25, 2.5, and 5 μM, chosen accordingly with the aforementioned report, resulted in eIF2α phosphorylation. We found that rafoxanide induced eIF2α phosphorylation, without affecting the basal eIF2α expression, in both HCT-116 and DLD1 cell lines (Figure 1A and Figure S1). Consistent with such results, flow-cytometry analysis on living cells (Figure S2) showed that rafoxanide treatment increased the percentage of ecto-CALR expressing HCT-116 and DLD1 cells (Figure 1B).

### 2.2. CRC Cells Release ATP and HMGB1 after Rafoxanide Exposure

**Figure 2:**
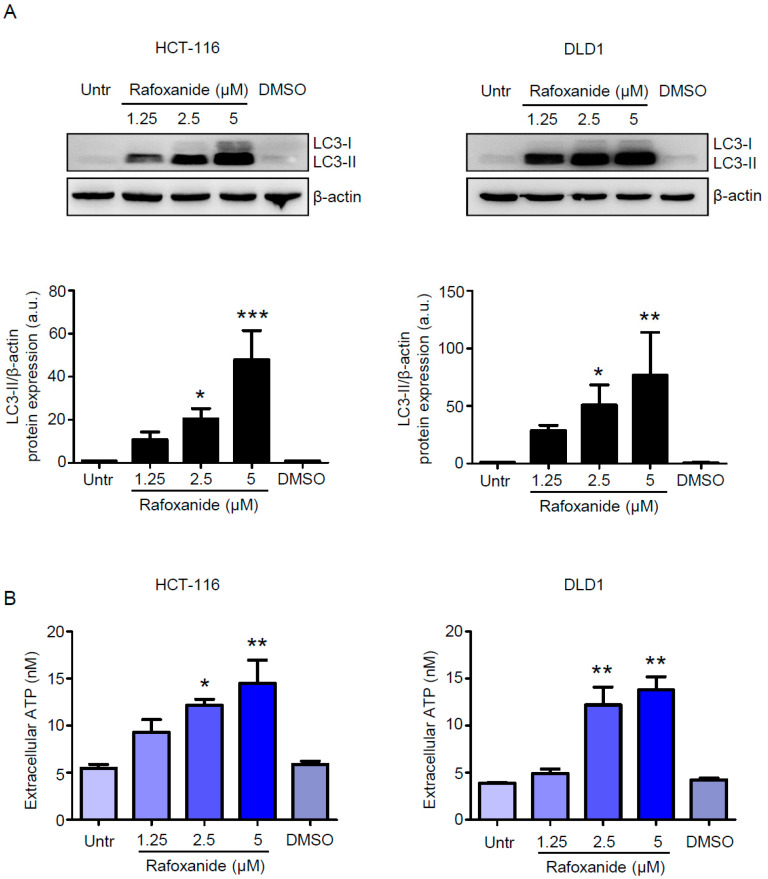
Rafoxanide induces autophagy and ATP release in CRC cells. (**A**) Western blotting for LC3 in extracts of HCT-116 and DLD1 cells either left untreated (Untr) or treated with either DMSO (vehicle) or rafoxanide for 24 h. β-actin was used as loading control. The full blots are available in Figure S3 from Supplementary Materials. One of three experiments in which similar results were obtained is shown. Lower insets: Quantitative analysis of LC3-II/β-actin protein ratio in total extracts of HCT-116 and DLD1 as measured by densitometry scanning of Western blots. Values are expressed in arbitrary units (a.u.) and are the mean ± SD of three experiments. Data were analyzed using one-way analysis of variance (ANOVA) followed by Dunnett’s post hoc test. DMSO vs. rafoxanide-treated cells, * *p* < 0.05, ** *p* < 0.01, *** *p* < 0.001. (**B**) Histograms showing the amount of released ATP in the medium supernatant of HCT-116 and DLD1 cells either left untreated (Untr) or treated with either DMSO or rafoxanide for 24 h. Data are expressed as mean ± SD of three experiments. Data were analyzed using one-way analysis of variance (ANOVA) followed by Dunnett’s post hoc test. DMSO vs. rafoxanide-treated cells, * *p* < 0.05, ** *p* < 0.01.

Another indication of ICD is the release of ATP during the pre-apoptotic or early/mid-apoptotic phases of cell death [26]. ATP acts as a chemoattractant for DC precursors expressing purinergic receptors [27]. As pre-mortem autophagy is required for the ICD-associated secretion of ATP [28], we first evaluated whether rafoxanide treatment could induce autophagy in CRC cells. The microtubule-associated protein light chain 3 (LC3) is commonly used to monitor autophagy [29]. During the autophagic process, the soluble form of LC3 (LC3-I) is conjugated to phosphatidylethanolamine. The resulting LC3-phosphatidylethanolamine complex, termed LC3-II, is tightly bound to autophagosomal membranes and LC3-II increase is considered one of the autophagy hallmarks [29]. Thus, we evaluated the autophagic process by assessing LC3-II accumulation. Rafoxanide markedly increased the protein levels of LC3-II at the concentrations tested (Figure 2A and Figure S3).

**Figure 3:**
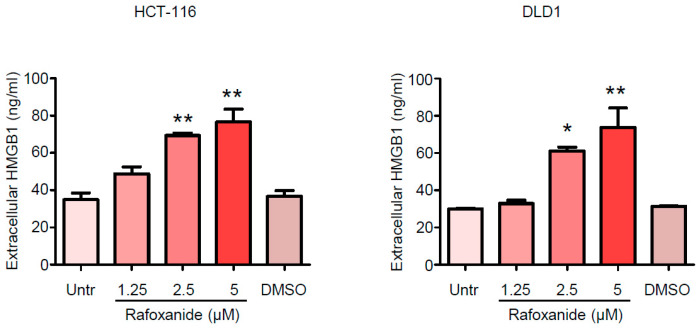
Rafoxanide induces HMGB1 release in CRC cells. Representative histograms showing the amount of released HMGB1 in the medium supernatant of HCT-116 and DLD1 cells either left untreated (Untr) or treated with either DMSO (vehicle) or rafoxanide for 48 h. Data are expressed as mean ± SD of two experiments. Data were analyzed using one-way analysis of variance (ANOVA) followed by Dunnett’s post hoc test. DMSO vs. rafoxanide-treated cells, * *p* < 0.05, ** *p* < 0.01.

Such observation is in line with the evidence reported by Liu et al., which shows that rafoxanide significantly promoted LC3-II accumulation and the formation of autophagic vacuoles in gastric cancer cells [17]. Consistently, we demonstrated that exposure of HCT-116 and DLD1 cells to rafoxanide for 24 h—a time point that does not affect the viability of such cells as previously reported [21]—provoked the release of ATP into the extracellular space (Figure 2B). HMGB1 is a non-histone chromatin-binding protein localized in the nucleus, where it interacts with DNA and regulates transcription [30]. The translocation of HMGB1 from the nucleus to the cytoplasm and its secretion or passive release through the permeabilized plasma membrane of succumbing/dead cells constitutes a major cellular danger signal and hallmark of ICD [30]. Indeed, extracellular HMGB1 interacts with Toll-like receptor-4 to stimulate the antigen-presenting function of maturing DCs [31]. At 48 and 60 h respectively, we found significantly increased levels of HMGB1 in the supernatants from rafoxanide-treated HCT-116 and DLD1 cells (at doses of 2.5 and 5 μM), as compared with vehicle (DMSO) (Figure 3).

Taken together, our data demonstrated that rafoxanide treatment induced key ICD-related markers in human CRC cells.

### 2.3. Anti-Cancer Effects of Rafoxanide on the Murine Adenocarcinoma Cell Line CT26 Associate with the Induction of ICD Markers

**Figure 4:**
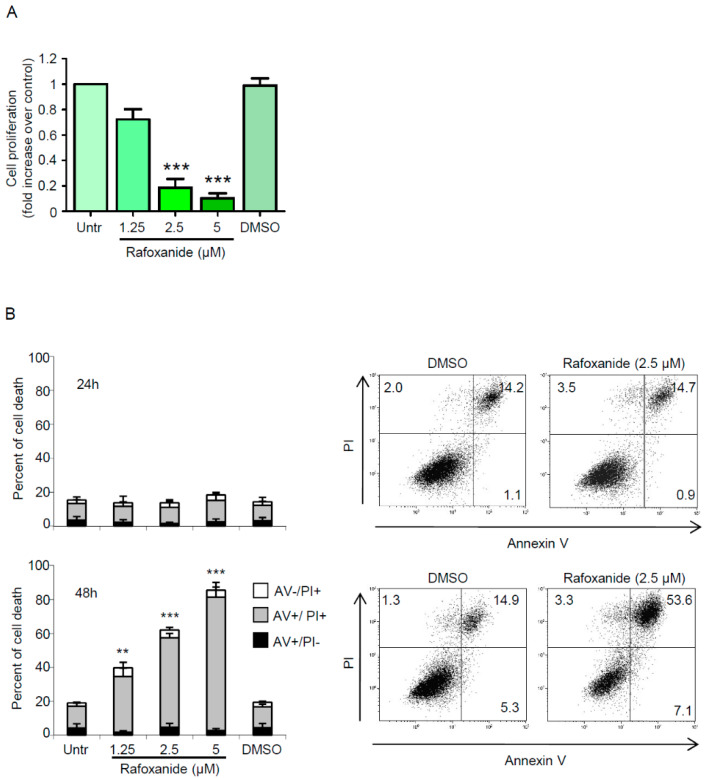
Rafoxanide inhibits proliferation and induces cell death in the murine colon adenocarcinoma cell line CT26. (**A**) CT26 cells were either left untreated (Untr) or treated with rafoxanide or DMSO (vehicle) for 24 h. Cell growth was assessed through a BrdU assay. Data indicate mean ± SEM of three experiments. Data were analyzed using one-way analysis of variance (ANOVA) followed by Dunnett’s post hoc test. DMSO vs. rafoxanide-treated cells: *** *p* < 0.001. (**B**) Histograms showing the percentage of cell death in CT26 cells either left untreated (Untr) or treated with either DMSO or rafoxanide for 24 h (upper panel) and 48 h (lower panel). Cell death was assessed by flow-cytometry analysis of Annexin V (AV) and/or propidium iodide (PI)-positive cells. Data are expressed as mean ± SD of three experiments. Data were analyzed using ANOVA followed by Dunnett’s post hoc test. DMSO vs. rafoxanide-treated cells, ** *p* < 0.01, *** *p* < 0.001. Right insets. Representative dot-plots showing the percentages of AV- and/or PI-positive cells treated with either DMSO or rafoxanide for 24 h (upper panel) and 48 h (lower panel).

Despite their undisputable convenience in the preliminary assessment of potential ICD inducers, ex vivo experiments cannot substitute for vaccination tests in vivo, as some agents are efficient in eliciting all the ICD hallmarks when administered to cancer cells in vitro, and yet those cells are unable to initiate anti-cancer immunity [8]. Actually, the gold-standard approach for identifying whether a specific stimulus is an ICD inducer relies on in vivo vaccination experiments using immunocompetent mice and syngeneic cancer cells [32]. Thus, to experimentally test our hypothesis that rafoxanide was able to efficiently trigger ICD, we employed the BALB/c-derived colon adenocarcinoma cell line CT26. We initially showed that rafoxanide significantly reduced the proliferation of cultured CT26 cells (Figure 4A).

**Figure 5:**
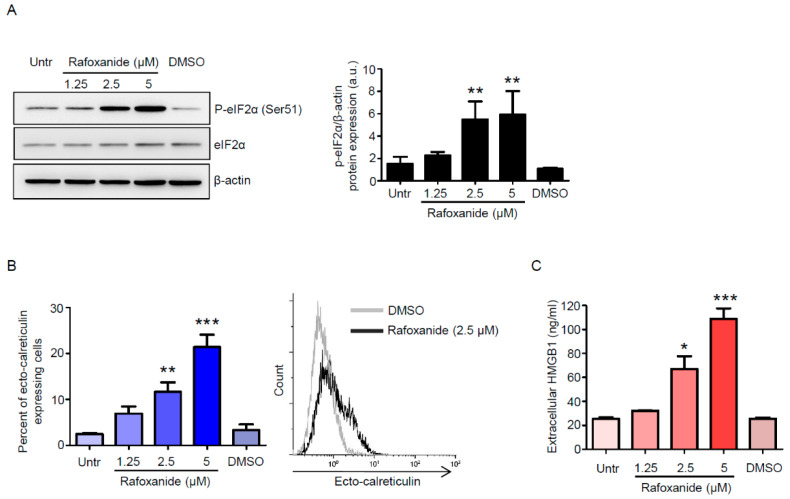
Rafoxanide induces immunogenic cell death markers in CT26 cells. (**A**) Representative western blotting for p-eIF2α (Ser51) and eIF2α in extracts of CT26 cells either left untreated (Untr) or treated with either DMSO (vehicle) or rafoxanide for 30 min. β-actin was used as loading control. The full blots are available in Figure S4 from Supplementary Materials. One of three experiments where similar results were obtained is shown. Right inset: Quantitative analysis of p-eIF2α (Ser51)/β-actin protein ratio in total extracts of CT26 as measured by densitometry scanning of Western blots. Values are expressed in arbitrary units (a.u.) and are the mean ± SD of three experiments. Data were analyzed using one-way analysis of variance (ANOVA) followed by Dunnett’s post hoc test. DMSO vs. rafoxanide-treated cells, ** *p* < 0.01. (**B**) Histograms showing the percentage of ecto-calreticulin-expressing CT26 cells either left untreated (Untr) or treated with either DMSO or rafoxanide for 6 h. Results indicate the percentage of ecto-calreticulin-expressing cells as assessed by flow-cytometry analysis. Data are expressed as mean ± SD of three experiments. Data were analyzed using one-way analysis of variance (ANOVA) followed by Dunnett’s post hoc test. DMSO vs. rafoxanide-treated cells, ** *p* < 0.01, *** *p* < 0.001. Right inset. Histograms showing ecto-calreticulin in CT26 treated with either DMSO or rafoxanide as assessed by flow-cytometry. (**C**) Amount of released HMGB1 in the medium supernatant of CT26 cells either left untreated (Untr) or treated with either DMSO or rafoxanide for 48 h. Data are expressed as mean ± SD of two experiments. Data were analyzed using ANOVA followed by Dunnett’s post hoc test. DMSO vs. rafoxanide-treated cells, * *p* < 0.05, *** *p* < 0.001.

We next investigated whether prolonged treatment with rafoxanide influenced CT26 cell survival. CT26 cells were treated with rafoxanide up to 48 h, and cell death was then evaluated by flow cytometry. No significant change in the percentage of AV+ and/or PI+ cells was seen after 24-hr treatment with rafoxanide (Figure 4B). However, analysis at 48 h revealed that rafoxanide strongly increased the percentage of dying/dead cells (as assessed by the frequency of AV+ and/or PI+ cells) (Figure 4B). Such results indicate that the rafoxanide-driven cell death was secondary to cell growth inhibition, in line with data previously reported for human CRC cells [21]. Later, we investigated whether CT26 cells expressed/released ICD markers following rafoxanide exposure. We showed that the drug promoted eIF2α Ser51 phosphorylation (Figure 5A and Figure S4) as well as the pre-apoptotic cell surface exposure of CALR (Figure 5B). Analysis of the cell-free supernatants after 48 h treatment with rafoxanide revealed more HMGB1 protein as compared with the ones harvested from DMSO-treated cells (Figure 5C).

### 2.4. Rafoxanide Exerts Protective Anti-Cancer Activity in a Vaccination Assay

**Figure 6:**
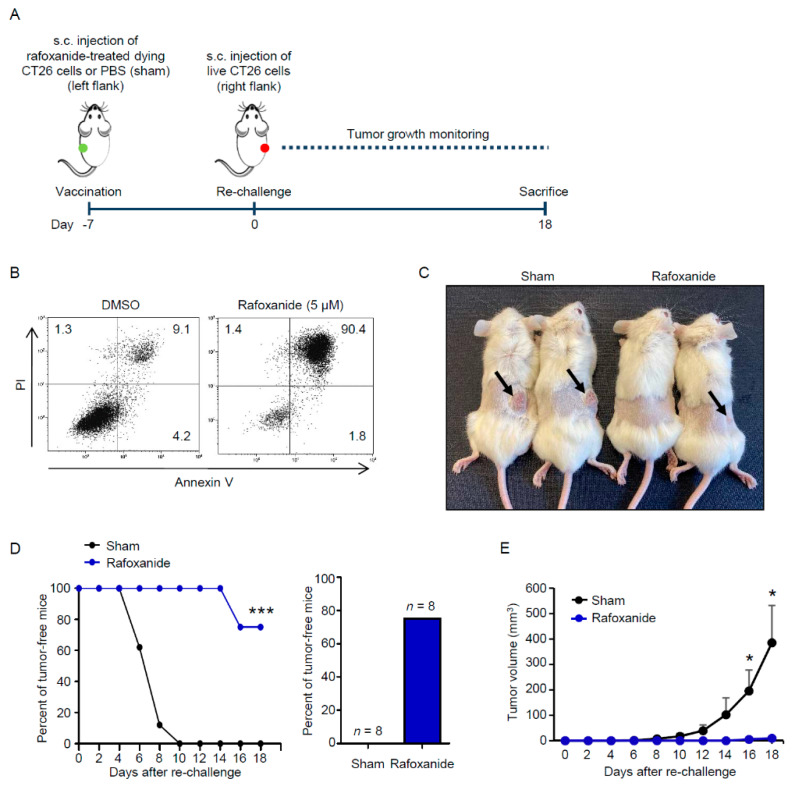
Rafoxanide has anti-cancer vaccination effect on CRC. (**A**). Experimental protocol of the tumor vaccination model. CT26 cells were treated with 5μM rafoxanide for 48 h. Then the cells were collected and subcutaneously (s.c.) injected (1 × 10^6^ cells per mouse) into the left flank of immunocompetent BALB/c mice (vaccination). An equal volume of PBS was used as control (sham). One week later (day 0), all mice were re-challenged with living CT26 cells (1 × 10^6^ per mouse) into the right flank to test the elicitation of immunological memory. Tumor growth was monitored until sacrifice (day 18). (**B**). Representative dot-plots showing the percentages of AV- and/or PI-positive CT26 cells treated with either DMSO (vehicle) or 5 μM rafoxanide for 48 h. Rafoxanide-treated CT26 cells were collected and used for in vivo vaccination as indicated in A. (**C**). Representative images of immunocompetent BALB/c mice vaccinated with either rafoxanide-treated succumbing CT26 cells or PBS (sham) and then re-challenged with living CT26 cells as indicated in A. Mice were sacrificed 18 days after the re-challenge. Arrows indicate CT26-derived tumors growing at the re-challenge site. (**D**). Evolution of tumor incidence over time as a Kaplan–Meier curve in mice treated as indicated in A. Shown is the percentage of tumor-free mice pooled from 2 independent experiments (*n* = 8 mice per group). Statistical significance was calculated by log-rank (Mantel-Cox) test. *** *p* < 0.001, as compared to mice vaccinated with PBS (sham). The right inset indicates the percentage of mice that were tumor-free at the end of the protocol (day 18). Data are derived from two independent experiments and the number of animals globally employed for these determinations is indicated. (**E**). Evolution of tumor growth over time. Tumor volume is represented as mean + SD. Statistical significance was calculated using the two-tailed Student’s *t*-test. * *p* < 0.05, as compared to mice vaccinated with PBS (sham).

Finally, we studied the immunogenic potential of rafoxanide in a vaccination setting. We treated CT26 cells in vitro with 5 μM rafoxanide for 48 h and injected them into the left flank of immunocompetent BALB/c mice (Figure 6A).

In parallel, an aliquot of rafoxanide-treated CT26 cells was assessed for cell viability. Flow cytometry analysis showed that the percentage of rafoxanide-treated succumbing/dead CT26 cells was more than 90% (Figure 6B). One week later, the mice were re-challenged with living CT26 cells injected into the contralateral flank to test the elicitation of immunological memory (Figure 6A). Mice were then monitored for tumor growth until sacrifice (day 18). We observed increased tumor-free survival among mice immunized with rafoxanide-treated tumor cells as compared with the sham (Figure 6C,D). At the end of the experiment, all the mice in the sham group had developed macroscopically and palpable tumors (Figure 6C,E). In contrast, among the animals vaccinated with rafoxanide-treated CT26 cells, 6 out of 8 mice (75%) had no visible sign of tumor growth and 2 out of 8 mice (25%) presented only tiny masses (having a volume of 6 and 13.5 mm^3^ respectively) at the site of re-challenge (Figure 6C–E).

## 3. Discussion

Conventional anti-cancer chemotherapy or radiotherapy is generally thought to reduce tumor progression by direct cytostatic/cytotoxic effects on tumor cells. However, emerging evidence suggests that the clinical success of conventional chemotherapy does not merely rely on cell toxicity, but also results from the restoration of tumor immune surveillance [33]. Anti-tumor immune response can be primed by ICD, a type of cell death characterized by cell-surface translocation of CALR, extracellular release of ATP and HMGB1. In support of this view are results derived from CRC patients treated with anthracyclines or oxaliplatin, known to promote ICD, demonstrating that favorable clinical outcomes associated with an increased number of cytotoxic CD8+ T cells within the tumor [34,35,36], whereas loss of DC function was a negative predictor of the therapeutic response to such agents in both clinical and preclinical settings [37,38].

In this study we aimed at investigating whether rafoxanide might induce ICD of CRC cells. We found that rafoxanide induced eIF2α phosphorylation in HCT-116 and DLD1 cells, and this was associated with an increased percentage of ecto-CALR expressing cells. ATP release, which acts as a chemoattractant for DC precursors expressing purinergic receptors [39], and secretion of HMGB1, which serves as a DC maturation factor by activating Toll-like receptor-4 [31], occur in the pre-apoptotic or early/mid-apoptotic phases and at the late stage of cell death, respectively. As pre-mortem autophagy is required for the ICD-associated secretion of ATP [28], we first evaluated whether rafoxanide treatment could induce autophagy in CRC cells. We found that rafoxanide triggered the autophagic process in both HCT-116 and DLD1 cells, as demonstrated by the marked LC3-II accumulation, considered one of the major hallmarks of autophagy [29]. Consistently, we demonstrated that rafoxanide treatment of CRC cells provoked the release of ATP into the extracellular space. In addition, we reported a marked increase of HMGB1 in the supernatants of rafoxanide-treated CRC cells. These observations indicated a potential ICD scenario in CRC cells following rafoxanide exposure and were congruent with the ability of other anti-helmintic agents to induce ICD hallmarks (i.e., ivermectin) [40] and cellular release of HMGB1 (e.g., albendazole, mebendazole, oxibendazole) [41].

To experimentally test our hypothesis that rafoxanide was an ICD inducer in vivo, we employed the BALB/c-derived colon adenocarcinoma cell line CT26 in a vaccination setting. Initially, we showed that rafoxanide provoked CT26 cell death as well as the induction of ICD-related markers, consistently with the data obtained with human CRC cells. Pre-treatment of immunocompetent BALB/c mice with rafoxanide-treated succumbing CT26 cells greatly suppressed the subsequent growth of CT26-derived tumors and increased tumor-free survival, indicating the establishment of a productive anti-tumor immune response. Future experimental efforts will be aimed at addressing the effects of rafoxanide in a therapeutic setting by treating tumor bearing mice with the drug directly to see whether an immune response is provoked against existing tumors.

The precise mechanism/s underlying the rafoxanide-driven ICD remains to be determined. In this context, it is worth mentioning a recent study showing that the specific CDK4/CDK6 inhibitor Abemaciclib promoted anti-tumor immunity through ICD induction [42]. As rafoxanide was proposed (through molecular docking studies) as a dual CDK4/CDK6 inhibitor [18], it is tempting to speculate this may be the mechanism or one of the mechanisms by which the drug promotes ICD.

Based on their ability to promote cancer cell death and release DAMPs, ICD inducers are divided into two categories, namely, type I and type II inducers [43]. The former, which enclose the majority of known ICD inducers, elicit immunogenicity via complementary ERS, in parallel with the main ERS-targeted effect that sparks apoptosis. The latter, instead, selectively target the endoplasmic reticulum and affect both danger and CHOP-mediated apoptotic signaling through a focused (ROS-based) ERS response [44]. Several pieces of evidence showing that rafoxanide is able to target multiple oncogenic pathways (e.g., CDK4/6, BRAF, PTEN/PI3K/Akt) [17,18,19,20,21] plead in favor of the idea that the drug is a Type I ICD inducer. Future in-depth experimental work will be however needed to ascertain the role of ROS and/or CHOP in the rafoxanide-driven ICD and confirm this hypothesis.

A possible hurdle to the application of rafoxanide as an ICD inducer in the clinic is that ICD alone is unlikely to entirely subvert the highly immunosuppressive tumor microenvironment [44]. In this context, combinatorial approach of ICD inducers (e.g., radiotherapy or anthracyclines) with immune-checkpoint blockers have been proven to have superior benefit in preclinical models [45] and cancer patients [46]. Thus, future experimental efforts should be aimed at assessing whether rafoxanide is useful in increasing tumor cell susceptibility to well-established immunotherapeutics, such as monoclonal antibodies that target immune checkpoints (e.g., programmed cell death-1 (PD-1), cytotoxic T-lymphocyte-associated protein-4 (CTLA-4)). In support of this expectation are recent studies showing that the anti-helmintic drug niclosamide and the CDK inhibitor dinaciclib enhanced the efficacy of PD-1/PD-L1 immune checkpoint blockade in non-small cell lung cancer and colon adenocarcinoma, respectively [47,48].

Another issue to the application of ICD in the clinic is that, while being effective in killing cancer cells, therapeutics tend to also kill the immune cells, thus weakening the ICD-driven host immune response, although a transient lymphodepletion was suggested to be beneficial due to the elimination of potential pro-tumorigenic/tumor suppressive immune cells [49]. Our preliminary experiments suggest that rafoxanide (when used at the same concentrations and for the same time-points reported in his study) has no apparent toxicity on human peripheral blood mononuclear cells (personal unpublished observations). However, studies aimed at assessing the effects of rafoxanide on different immune cell subsets isolated by the colonic mucosa are necessary and currently ongoing, as we cannot rule out the possibility that rafoxanide may affect viability and/or cytokine production and/or activity of such cells.

## 4. Materials and Methods

### 4.1. Cell Culture

All reagents were from Sigma-Aldrich (Milan, Italy), unless specified. The human CRC cell lines HCT-116 was obtained from the American Type Culture Collection (ATCC, Manassas, VA, USA) and maintained in McCoy’s 5A medium supplemented with 10% fetal bovine serum (FBS) and 1% penicillin/streptomycin (P/S) (Lonza, Verviers, Belgium). The human CRC cell line DLD1 and the murine colon adenocarcinoma cell line CT26 were obtained from ATCC and maintained in RPMI 1640 medium supplemented with 10% FBS and 1% P/S. Cell lines were recently authenticated by STR DNA fingerprinting using the PowerPlex 18D System kit according to the manufacturer’s instructions (Promega, Milan, Italy). The STR profiles of all the cell lines matched the known DNA fingerprints. To determine whether rafoxanide induced eIF2α phosphorylation and LC3-II accumulation, cells were either left untreated or treated with either rafoxanide or dimethyl sulfoxide (DMSO) for 30 min and 24 h, respectively.

### 4.2. Western Blotting

Protein extracts were prepared and run as described elsewhere [50]. Blots were incubated with antibodies against p-eIF2α (Ser51) (#3597, 1:1000 final dilution, Cell Signaling Technology, Danvers, MA, USA), eIF2α (sc-11386, 1:1000 final dilution, Santa Cruz Biotechnology, Inc., Dallas, TX, USA), LC3B (#2775, 1:1000 final dilution, Cell Signaling Technology), followed by a secondary antibody conjugated to horseradish peroxidase (P0161 or P0448, 1:20,000 final dilution, Dako, Santa Clara, CA, USA). After analysis, each blot was stripped and incubated with a mouse-anti-human monoclonal β-actin antibody (A544, 1:5000 final dilution), followed by a secondary antibody conjugated to horseradish peroxidase (P0161, 1:20,000 final dilution, Dako) to ascertain equivalent loading of the lanes. Densitometry analysis was performed using Image Lab Software 5.2.1 (Bio-Rad Laboratories, Inc., Hercules, CA, USA).

### 4.3. Assessment of ecto-CALR, ATP and HMGB1

To assess whether rafoxanide induced CALR exposure on the cell surface, HCT-116, DLD1, and CT26 cells were either left untreated or treated with either rafoxanide or DMSO for 6 h. Cells were then harvested, washed with FACS buffer (1 × PBS, 5% FBS, and 0.1% sodium azide), and incubated with a rabbit anti-CALR antibody (1:100, PA3-900, Thermo Fisher Scientific, Rome, Italy) in FACS buffer at 4 °C for 30 min. Cells were then washed and incubated with a FITC-conjugated secondary antibody (1:500) in FACS buffer at 4 °C for 30 min. After washing two times with FACS buffer, surface CALR was detected by Gallios flow cytometer (Beckman Coulter, Milan, Italy). In parallel, cells were stained with a control isotype primary antibody. To test whether rafoxanide induced the release of ATP, 2 × 10^5^ HCT-116 and DLD1 cells were plated in 6-well plates with 1.5 mL full medium appropriate for the cell types. The medium was changed 24 h later and the cells were either left untreated or treated with either rafoxanide or DMSO. After 24 h, cell-free supernatants were collected. Extracellular ATP was determined by the luciferase/luciferin-based ENLITEN ATP Assay (Promega), following the manufacturer’s instructions. Chemiluminescence was measured on a GloMax^®^-Multi Detection System (Promega). For detection of HMGB1 release, 2 × 10^5^ HCT-116, DLD1 and CT26 cells were plated in 6-well plates with 1.5 mL full medium appropriate for the cell types. The medium was changed 24 h later and cells were either left untreated or treated with either rafoxanide or DMSO. After 48 h, cell-free supernatants were collected. Quantification of HMGB1 in the supernatants was assessed by enzyme-linked immunosorbent assay (IBL International GmbH, Hamburg, Germany) according to the manufacturer’s instructions.

### 4.4. Assessment of Cell Proliferation and Death

Cell proliferation was assessed by using a commercially available BrdU assay kit (Roche Diagnostics, Monza, Italy). Briefly, 5000 CT26 cells were cultured in 96-well microplates and allowed to adhere overnight. Cells were either left untreated or treated with rafoxanide or DMSO for 24 h. BrdU was added to the cell cultures 6 h before the end of the treatments and cell growth was evaluated by ELISA. To score cell death, HCT-116, DLD1, and CT26 cells were either left untreated or treated with rafoxanide or DMSO for 6–48 h. Cells were then collected, washed twice in PBS, stained with FITC-annexin V (AV, 1:100 final dilution, Immunotools, Friesoyte, Germany) according to the manufacturer’s instructions and incubated with 5 μg/mL PI for 30 min at 4 °C. Fluorescence was measured using a Gallios (Beckman Coulter, Milan, Italy) flow cytometer. Viable cells were considered as AV−/PI− cells, apoptotic cells as AV+/PI− cells, while secondary necrotic cells were characterized by AV+/PI+ positive staining.

### 4.5. Tumor Vaccination Assay

BALB/c mice were obtained from the Charles River Laboratories (Lodi, Italy) and maintained in filter-topped cages on autoclaved food and water at the University of Rome “Tor Vergata” animal facility (Rome, Italy). All animal experiments were approved by the local Institutional Animal Care and Use Committee (authorization 494/2017-PR, registered with the Italian Ministry of Health). Rafoxanide-treated CT26 cells were collected, washed twice with PBS, and subcutaneously (s.c.) injected (1 × 10^6^ cells per mouse in 100 μL PBS) into the left flank of immunocompetent eight-week-old female BALB/c mice (vaccination). An equal volume of PBS was used as control (sham). One week later (day 0), all mice were re-challenged with living CT26 cells (1 × 10^6^ per mouse in 100 μL PBS) into the right flank to test the elicitation of immunological memory. Size of tumors was evaluated every other day with a caliper until sacrifice (day 18). Tumor volume was calculated using the formula: 0.5 × long diameter × short diameter^2^.

### 4.6. Statistical Analysis

Results were analyzed using the two-tailed Student’s t-test for comparison between two groups. Multiple comparison analysis was performed using one-way analysis of variance (ANOVA) followed by Dunnett’s post hoc test. Tumor-free survival in the vaccination assay was calculated by log-rank (Mantel-Cox) test. Significance was defined as *p*-values < 0.05.

## 5. Conclusions

Irrespective of the above-mentioned open questions, our data provide the first evidence that rafoxanide induces bona fide ICD, thus adding a new potential tool in the armamentarium of anti-cancer immunotherapy.

## Supplementary Materials

The following are available online at https://www.mdpi.com/2072-6694/12/5/1314/s1, Figure S1: Uncropped blots for p-eIF2α (Ser51), eIF2α, and β-actin representing those depicted in Figure 1A, Figure S2: Six h rafoxanide treatment does not affect HCT-116 and DLD1 cell survival, Figure S3: Uncropped blots for LC3 and β-actin representing those depicted in Figure 2A, Figure S4: Uncropped blots for p-eIF2α (Ser51), eIF2α, and β-actin representing those depicted in Figure 5A.
