# Supplementary material for: Rafoxanide Induces Immunogenic Death of Colorectal Cancer Cells
Source: Cancers (Basel). 2020 May 21;12(5):1314. doi: 10.3390/cancers12051314 (PMC7281008; doi:10.3390/cancers12051314)
Supplement: Supplementary file 1 [file cancers-12-01314-s001.pdf]

Supplementary Material: Rafoxanide induces Immunogenic Death of Colorectal Cancer Cells

Antonio Di Grazia, Federica Laudisi, Davide Di Fusco, Eleonora Franzè, Angela Ortenzi, Ivan Monteleone, Giovanni Monteleone and Carmine Stolfi

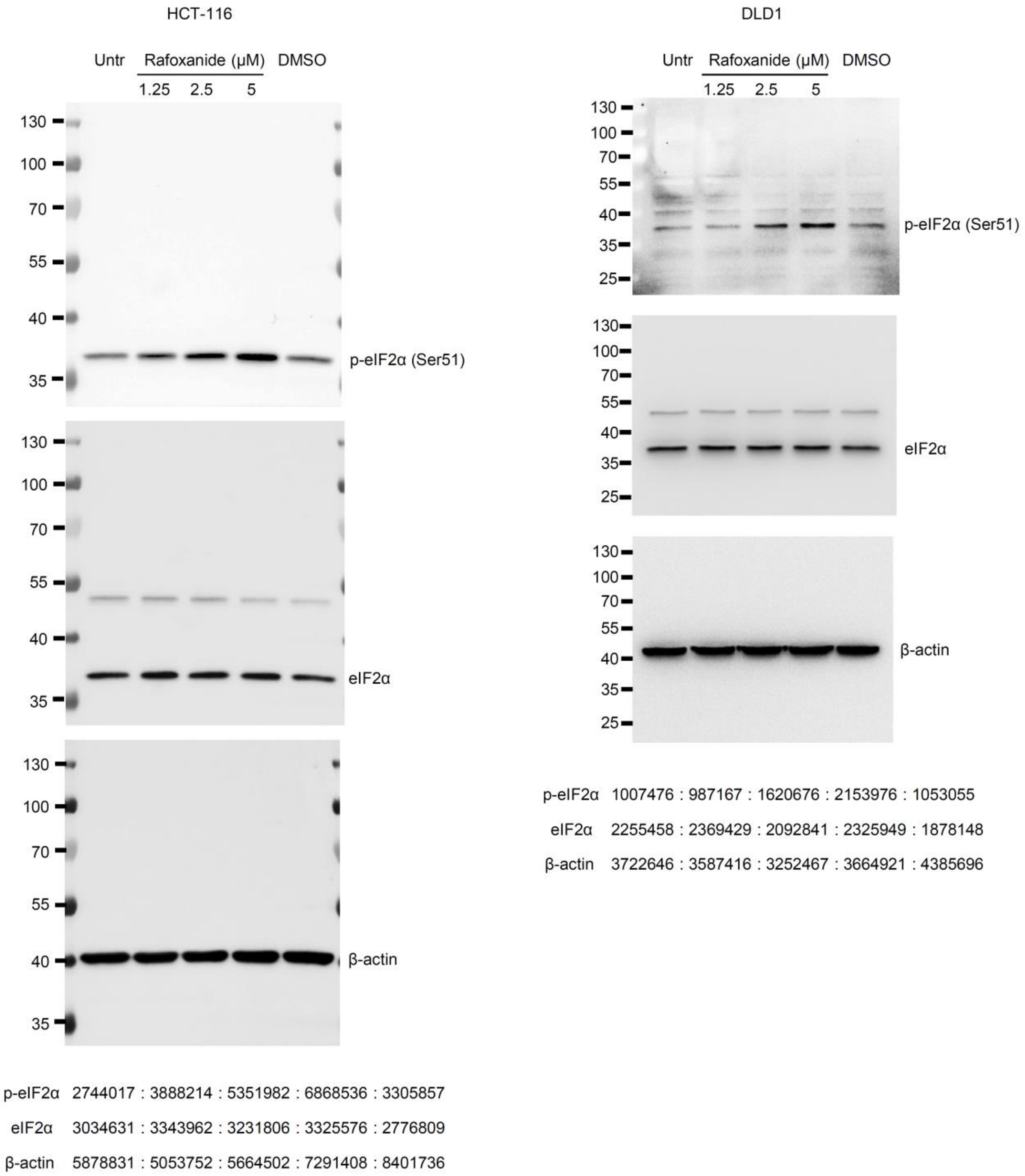

Figure S1. Uncropped blots for p-eIF2α (Ser51), eIF2α and β-actin representing those depicted in Figure 1A. Molecular weight markers and densitometry readings are indicated on the left and at the bottom, respectively.

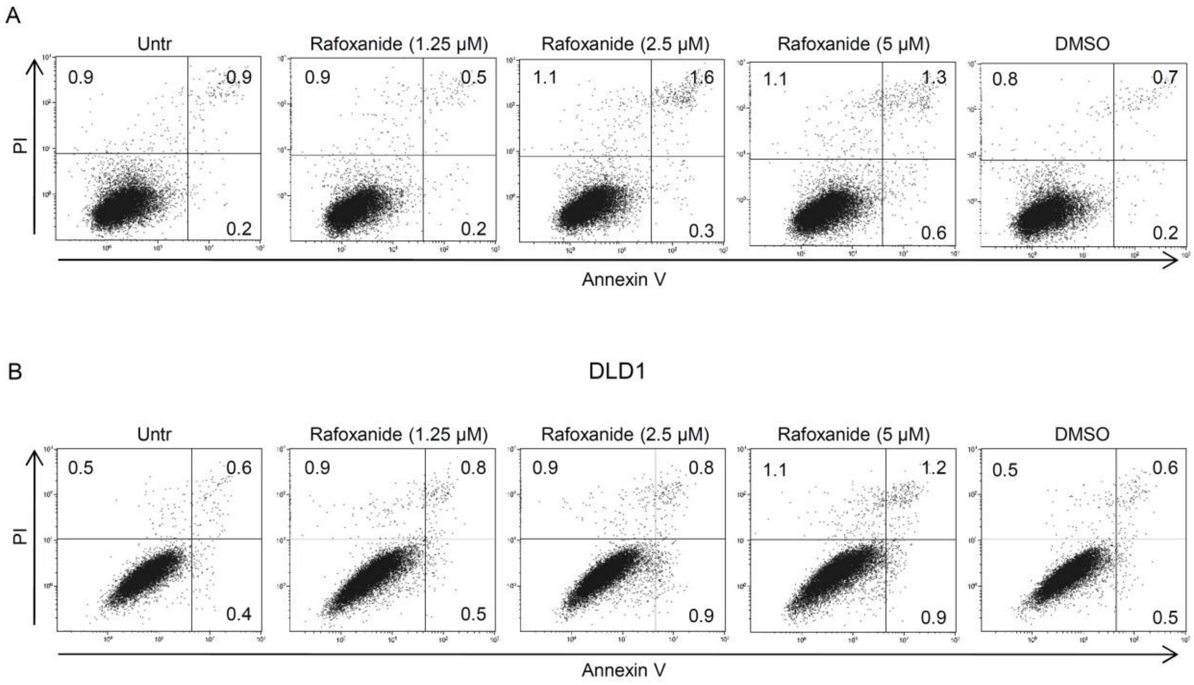

Figure S2. Six h rafoxanide treatment does not affect HCT-116 and DLD1 cell survival. HCT-116 (**A**) and DLD1 (**B**) cells were either left untreated (Untr) or treated with either DMSO (sham) or the indicated doses of rafoxanide for 6 h. Representative dot-plots show the percentages of AV- and/or PI-positive cells. One of three representative experiments in which similar results were obtained is shown.

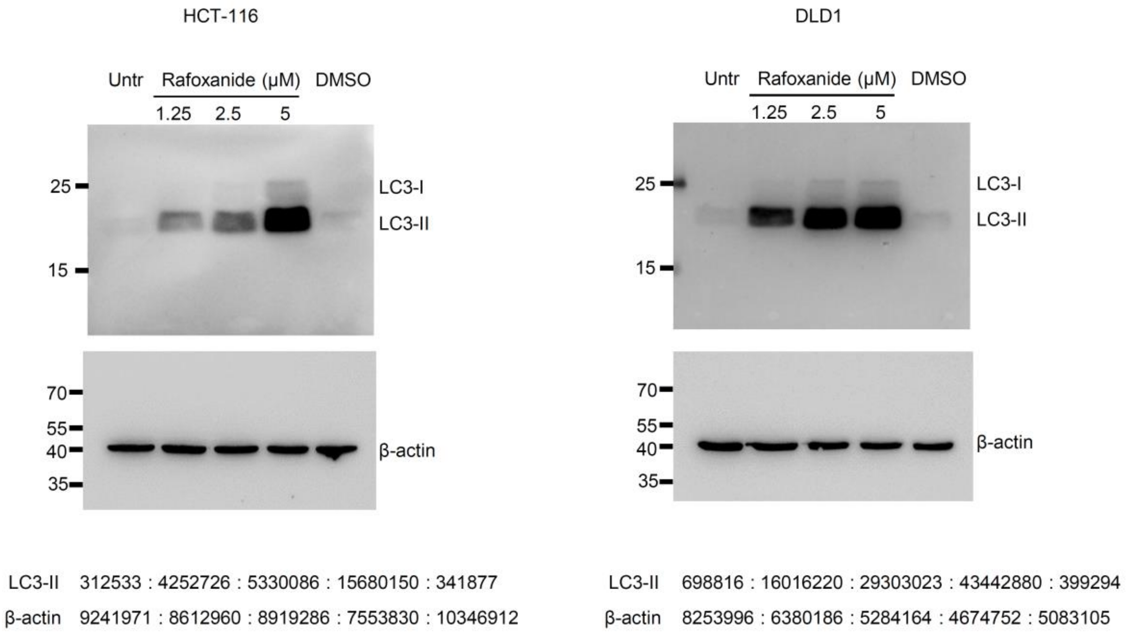

Figure S3. Uncropped blots for LC3 and β-actin representing those depicted in Figure 2A. Molecular weight markers and densitometry readings are indicated on the left and at the bottom, respectively.

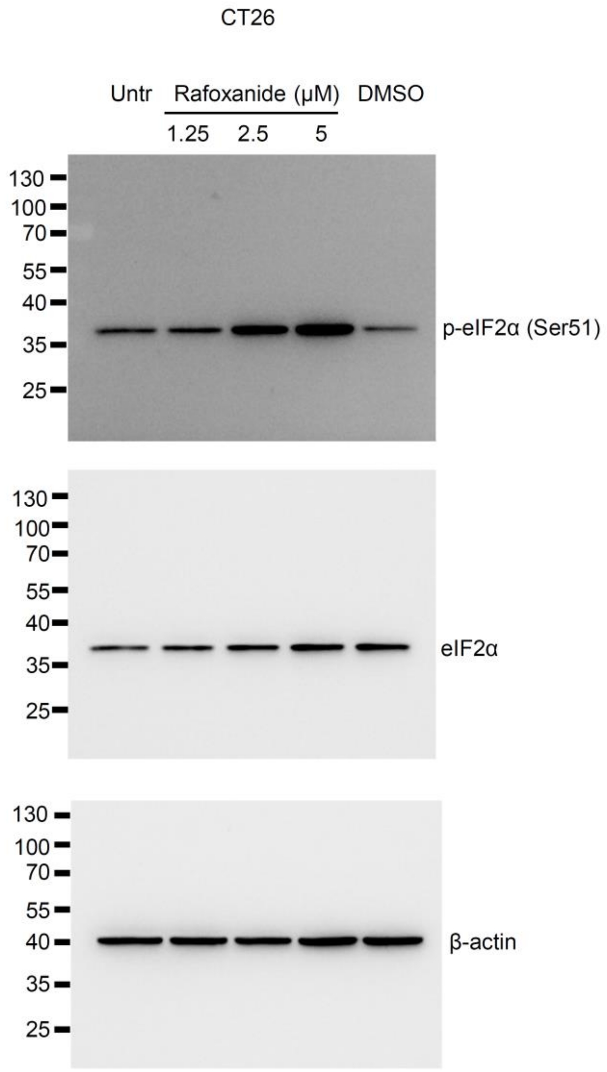

p-eIF2α 1632800 : 1699233 : 4593564 : 6826426 : 885222

eIF2α 7585228 : 8129924 : 9579480 : 11404852 : 11617737

β-actin 9851135 : 9222213 : 8604175 : 11367423 : 12062652

Figure S4. Uncropped blots for p-eIF2α (Ser51), eIF2α and β-actin representing those depicted in Figure 5A. Molecular weight markers and densitometry readings are indicated on the left and at the bottom, respectively.
